# Nitrogen and Sulfur Co-Doped Graphene-Quantum-Dot-Based Fluorescent Sensor for Rapid Visual Detection of Water Content in Organic Solvents

**DOI:** 10.3390/molecules29215178

**Published:** 2024-11-01

**Authors:** Hongyuan Zhang, Jieqiong Wang, Xiaona Ji, Yanru Bao, Ce Han, Guoying Sun

**Affiliations:** 1 School of Science, Changchun Institute of Technology, 395 Kuanping Road, Changchun 130012, China; lx_jxn@ccit.edu.cn (X.J.); 024baoyanru@163.com (Y.B.); 2 School of Materials Science and Engineering, Changchun University, 6543, Weixing Road, Changchun 130022, China; wangjq94@ccu.edu.cn; 3 State Key Laboratory of Electroanalytical Chemistry, Changchun Institute of Applied Chemistry, Chinese Academy of Sciences, Changchun 130022, China; 4 School of Chemistry and Life Science, Changchun University of Technology, 2055 Yanan Street, Changchun 130012, China

**Keywords:** fluorescent probes, graphene quantum dots, portable sensors, water content

## Abstract

Accurate water content detection is crucial for optimizing chemical reactions, ensuring product quality in pharmaceutical manufacturing, and maintaining food safety. In this study, nitrogen and sulfur co-doped graphene quantum dots (R-GQDs) were synthesized via a one-step hydrothermal method using o-phenylenediamine as the carbon source. The synthesis conditions, including reaction time, temperature, o-phenylenediamine concentration, and H_2_SO_4_/water ratio, were optimized using the Box-Behnken response surface methodology. The R-GQDs exhibited excellent fluorescence stability and distinct solvent-dependent characteristics, alongside a broad linear detection range and high sensitivity, making them highly suitable for dual-mode water content detection (colorimetric and fluorescent). To enhance the accuracy of visual detection, R-GQDs were incorporated into portable test strips with smartphone-assisted analysis, compensating for the human eye’s limitations in distinguishing subtle color changes. The sensor’s practical utility was validated through spiked recovery experiments in food samples, and the R-GQDs demonstrated good biocompatibility for in vivo imaging in shrimp. These findings highlight a novel strategy for developing portable, real-time water content sensors with potential applications in both portable detection systems and biological imaging.

## 1. Introduction

Graphene quantum dots (GQDs), as a novel class of carbon-based nanomaterials, have garnered significant attention due to their stable photoluminescence, high quantum yield, excellent biocompatibility, and cost-effective synthesis [1,2,3]. These properties render GQDs highly suitable for a range of applications, including fluorescent probes, bioimaging, light-emitting devices, and drug delivery [4,5]. However, one of the key limitations of conventional GQDs is their short-wavelength emission, which is readily absorbed by biological tissues and is susceptible to background interference, thereby restricting their efficacy in biosensing and imaging applications. To overcome these challenges, extensive research has been focused on doping and surface modification strategies to shift the emission wavelength towards the red or near-infrared (NIR) spectrum, thereby enhancing their utility for biological applications [6,7]. Nitrogen and sulfur co-doping, in particular, has emerged as a promising approach for fine-tuning the optical properties of GQDs due to the introduction of heteroatoms, which can modulate the electronic structure, improve charge distribution, and reduce non-radiative recombination, ultimately leading to enhanced fluorescence properties [8,9,10].

Fluorescent materials have seen significant advancements, and the ability to tune emission properties for different applications has become essential. The synthesis of multicolor fluorescent GQDs has emerged as a focal point in recent research, although the current methodologies remain complex and labor-intensive, often involving multiple processing steps [11]. Simplifying the synthesis by leveraging the influence of solvent polarity on GQD surface states would not only enable easier optical customization but also improve the efficiency and practicality of producing multicolor emissions.

Water, as a highly polar solvent, plays a critical role in various industrial processes, including organic reactions, pharmaceutical manufacturing, and food preservation. In organometallic chemistry, even trace amounts of water can drastically affect reaction efficiency, yield, and catalyst performance. Similarly, in pharmaceutical manufacturing and food safety, precise water content control is vital to ensure product stability, potency, and shelf life [12]. Traditional methods for detecting water content, such as Karl Fischer titration [13] and chromatography [14], although effective, involve the use of toxic reagents, non-portable equipment, and require specialized personnel. This necessitates the development of rapid, accurate, and cost-efficient alternatives for water content detection in organic solvents.

Recent advancements in fluorescent materials, including carbon dots [10], covalent organic frameworks [15], quinoline derivatives [16], boron dipyrromethene (BODIPY) [17] and fluorescent dyes [18] have been used in recent studies to detect water in organic solvents. For instance, Yang et al. developed solvent-dependent CDs for water detection in N,N-dimethylformamide [19], Cao et al. constructed hydrogen-bond-induced quinoline derivatives for monitoring water content in tetrahydrofuran (THF), acetonitrile, and dimethyl sulfoxide (DMSO) [16]. The integration of fluorescent sensors with smartphone-based platforms presents a more accessible, portable, and user-friendly solution compared to traditional methods, eliminating the need for complex instrumentation [20]. In light of these advancements, developing a smartphone-integrated, fluorescence-based, rapid-response sensor for detecting water content in organic solvents offers significant potential for applications in industrial and environmental monitoring.

**Scheme 1:**
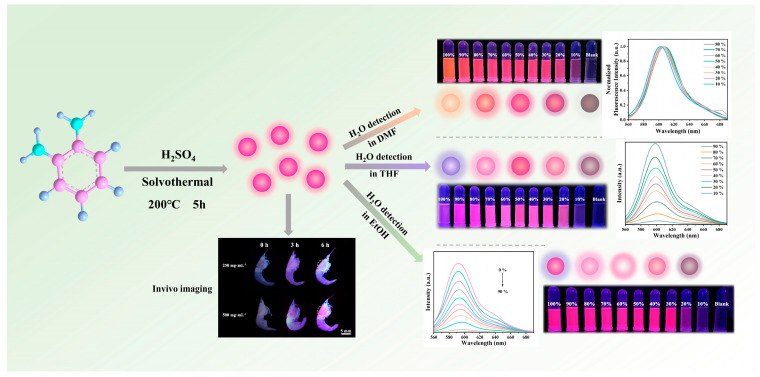
Synthesis of R-GQDs and schematic diagram of the sensor for detecting water content in organic solvents.

In this study, we synthesized sulfur and nitrogen co-doped graphene quantum dots (R-GQDs) with red emission using a one-step hydrothermal method, dispersing o-phenylenediamine in an H_2_SO_4_/water solution. R-GQDs exhibited a maximum emission peak at 602 nm under 536 nm excitation, with fluorescence intensity strongly correlated with water content in solution, demonstrating solvent-dependent characteristics (Scheme 1). R-GQDs were applied for detecting water content in solvents such as tetrahydrofuran (THF), N,N-dimethylformamide (DMF), and ethanol. The R-GQDs also displayed low background fluorescence, minimal cytotoxicity, and good biocompatibility, making them suitable for imaging in freshwater shrimp larvae. Additionally, R-GQDs were incorporated into test paper strips for visual detection of water content, with smartphone-based image capture and color analysis enabling a portable fluorescence sensing platform with a fast response time and wide detection range. In summary, this R-GQD-based portable sensing platform offers promising applications in water content detection and biosensing fields.

## 2. Results and Discussion

### 2.1. Synthesis of R-GQDs and Optimization of Reaction Conditions

R-GQDs have been synthesized using a one-step hydrothermal method with o-phenylenediamine in an H_2_SO_4_/water solution. To achieve optimal fluorescence emission intensity, a Box-Behnken Design (BBD) was employed to optimize four factors: the amount of o-phenylenediamine, reaction time, reaction temperature, and the volume ratio of H_2_SO_4_/water in the solution. Reaction time (X_1_), reaction temperature (X_2_), sulfuric acid/water volume ratio (X_3_), and o-phenylenediamine concentration (X_4_) were defined as independent variables, while the fluorescence emission intensity of the synthesized R-GQDs was designated as the response variable. High, medium, and low levels for each factor were established for the response surface experiments (Table S1), and the results from 29 experimental runs are presented in Table S2.

To further analyze and compare the experimental data, a second-order polynomial regression model has been developed to represent the fluorescence intensity of the synthesized R-GQDs as a function of the four factors, as shown in Equation (1):

(1) $$Y=746.39+2.93X_{1}-2.20X_{2}+3.86X_{3}-10.15X_{4}-14.42X_{1}X_{2}+\;0.44X_{1}X_{3}-2.62X_{1}X_{4}+8.20X_{2}X_{3}-12.15X_{2}X_{4}+3.29X_{3}X_{4}-\;47.67X_{1}^{2}-20.53X_{2}^{2}-34.67X_{3}^{2}-13.32X_{4}^{2}$$

In this model, the quadratic terms for X_1_ and X_3_ exhibit relatively large coefficients, indicating that reaction time and the H_2_SO_4_/water volume ratio are the primary factors influencing the fluorescence emission intensity of R-GQDs. As shown in Table S3, the analysis of variance (ANOVA) reveals that the lack-of-fit term (*p*-value) for the proposed model is less than 0.05, confirming its high significance in predicting changes in R-GQD fluorescence intensity [21]. Additionally, the *p*-values for $A_{1}^{2}$, $A_{2}^{2}$, and $A_{3}^{2}$ are all below 0.05, affirming their significant impact on fluorescence intensity during R-GQD synthesis [22].

Furthermore, as presented in Table S4, the model’s coefficient of determination (R^2^) is 0.9438, with an adjusted R^2^ value of 0.7376, indicating a strong correlation between the experimental data and the empirical model, thus validating the model’s effectiveness in predicting optimal experimental conditions. The coefficient of variation (CV%) for the model is 2.88%, demonstrating its reliability. The precision value, representing the signal-to-noise ratio, has been found to be 8.473 (greater than 4), indicating that the optimized experimental conditions based on this model possess sufficient precision to meet the anticipated experimental requirements [23].

Moreover, the reliability and applicability of the second-order model within the experimental range have been thoroughly analyzed. As illustrated in Figure S1a, there is a clear correlation between the actual data and the predicted values, demonstrating that the proposed model can effectively estimate the fluorescence intensity of the synthesized R-GQDs. Additionally, Figure S1b shows that the residuals follow a normal distribution concerning the predicted fluorescence intensity, further confirming the model’s validity.

### 2.2. Response Surface Analysis

To analyze the interactive effects of factors influencing R-GQD synthesis, 3D response surface plots are presented in Figure S2, clearly illustrating the interactions between factor pairs. During the analysis, two factors were held constant to reduce dimensionality and isolate the effects of the remaining two factors on the fluorescence intensity of R-GQDs.

In the contour plots, circular shapes indicate insignificant interactions, while elliptical shapes suggest significant interactions. The slope of the 3D plot reflects the strength of interactions; steeper slopes correspond to greater interactions. As shown in Figure S2a, the elliptical contour lines indicate a significant interaction between reaction time (A) and o-phenylenediamine concentration (D). Conversely, Figure S2b,c display more circular contours, suggesting that the interactions of reaction temperature (B) and H_2_SO_4_/water volume ratio (C) with reaction time (A) are less significant compared to those involving o-phenylenediamine concentration (D). A similar interaction pattern is evident in Figure S2d–f for other factors.

The factors influencing R-GQD synthesis have been ranked as follows: reaction time (A) > H_2_SO_4_/water volume ratio (C) > temperature (B) > o-phenylenediamine concentration (D), aligning with regression model analysis. The formation of R-GQDs involves high-temperature polymerization and carbonization, yielding GQDs with sp^2^ domains [24]. Prolonged high-temperature conditions lead to excessive dehydration and carbonization, causing fluorescence quenching due to non-radiative deactivation [25].

The H_2_SO_4_/water volume ratio plays a crucial role in introducing functional groups and heteroatom doping. The synergistic effects of doped N and S atoms modulate charge distribution, reducing non-radiative recombination and bandgap, thereby enhancing the photoluminescence of GQDs. However, excessive sulfuric acid can lower the pH excessively, inhibiting the condensation of o-phenylenediamine into R-GQDs [26]. Although temperature is not the primary factor, careful control is essential, as excessively high temperatures promote over-carbonization, leading to larger carbon structures and reduced efficiency.

Finally, the optimal conditions for the preparation of R-GQDs were determined to be: reaction time of 5 h, reaction temperature of 200 °C, H_2_SO_4_/water volume ratio of 1:19 (*v*/*v*), and o-phenylenediamine concentration of 230 mg.

### 2.3. Characterization of R-GQDs

**Figure 1:**
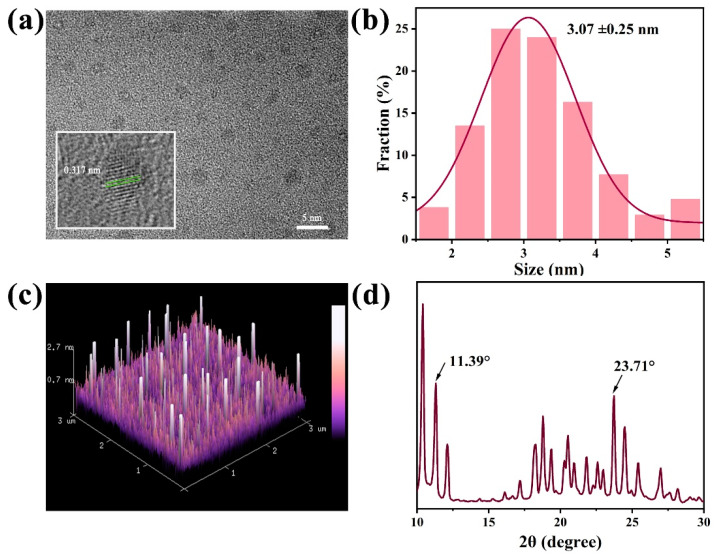
(**a**) TEM image of R-GQDs, inset is HR-TEM image; (**b**) diameter distribution of R-GQDs in aqueous solution, the black line is the Gaussian fitting curve; (**c**) AFM three-dimensional image of N-GQDs; (**d**) XRD spectrum of R-GQDs.

The morphology and size of the R-GQDs have been characterized by TEM, as shown in Figure 1a, revealing a quasi-spherical shape with a uniform distribution. HR-TEM analysis has indicated a lattice spacing of 0.317 nm, corresponding to the (0 0 2) plane of graphite. Particle size distribution analysis has shown an average diameter of 3.07 ± 0.25 nm (Figure 1b). Further AFM analysis (Figure 1c) has revealed an average height of 2.92 nm, consistent with the TEM results. The XRD pattern (Figure 1d) has displayed distinct peaks at 2θ = 11.39° and 23.71°, corresponding to the (0 0 2) and (1 0 0) planes of graphene, which was consistent with the results observed by HR-TEM, thereby confirming the successful synthesis of R-GQDs [27].

**Figure 2:**
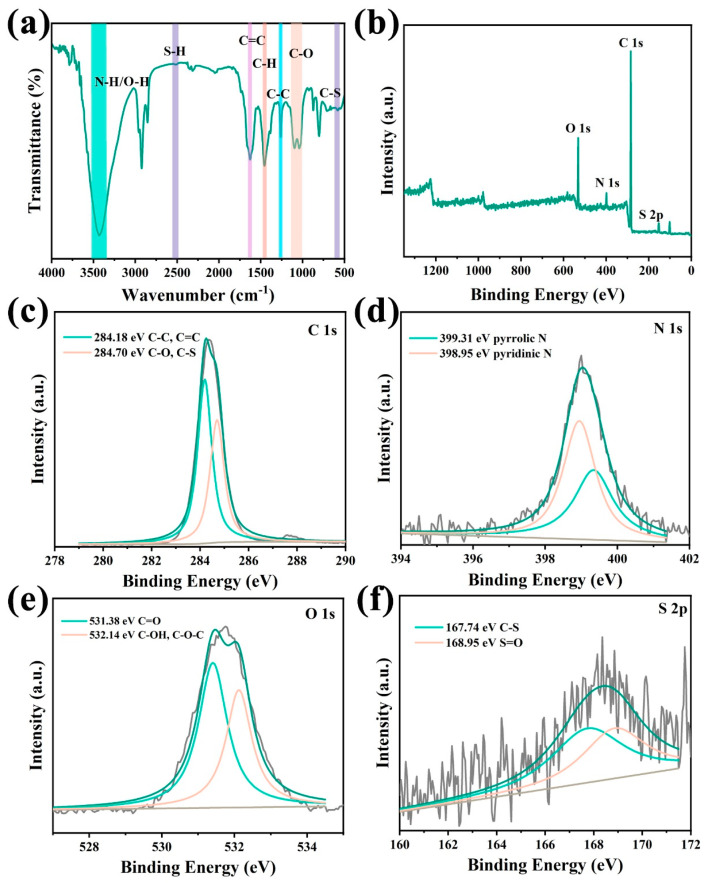
(**a**) FT-IR spectrum of R-GQDs; (**b**) XPS spectrum of R-GQDs and high-resolution spectra of (**c**) C 1s (The green line were C-C and C=C bonds, and the C-O and C-S bonds were represented by pink line, respectively), (**d**) N 1s (The green line was pyrrolic N, and the pyridinic was represented by pink line, respectively), (**e**) O1s (The green line was C=O bond, and the C-OH and C-O-C bonds were represented by pink line, respectively) and (**f**) S 2p (The green line was C-S, and the S-O bond was represented by pink line, respectively).

The surface functional groups of R-GQDs have been identified via Fourier-transform infrared (FT-IR) spectroscopy. As shown in Figure 2a, a broad absorption band at 3350 cm^−1^ has corresponded to N-H/O-H stretching vibrations, while the peak at 1636 cm^−1^ has been attributed to C=C stretching. A peak at 1328 cm^−1^ has indicated in-plane C-H bending, and weak peaks at 1044 cm^−1^, 1133 cm^−1^ and 1485 cm^−1^ have represented C-O, C-C and C-H stretching [28], respectively. Notably, peaks at 2546 cm^−1^ and 602 cm^−1^ have been linked to S-H and C-S stretching vibrations [29].

The elemental composition and surface functional groups have been further ana-lyzed via X-ray photoelectron spectroscopy (XPS). The full spectrum (Figure 2b) has shown four main elements: carbon (284.8 eV), nitrogen (398.9 eV), oxygen (530.9 eV), and sulfur (166.8 eV). High-resolution C 1s analysis (Figure 2c) has revealed peaks at 284.18 eV and 284.70 eV, corresponding to C-C/C=C and C-O/C-S bonds, respectively [30]. The N 1s spec-trum (Figure 2d) has displayed peaks at 399.31 eV and 398.95 eV, representing pyrrolic and pyridinic nitrogen. O 1s analysis (Figure 2e) has confirmed the presence of C=O, C-OH, and C-O-C groups with peaks at 531.38 eV and 532.14 eV. Lastly, the S 2p spectrum (Figure 2f) has shown peaks at 167.74 eV and 168.95 eV, indicative of C-S and S=O bonds [31], thereby confirming the incorporation of sulfur into the R-GQDs. Overall, FT-IR and XPS analysis confirm that nitrogen and sulfur have been successfully doped into R-GQDs and that their surfaces are rich in hydroxyl, amino, and thiol groups, which endow R-GQDs with excellent water solubility and stability.

The graphitization degree of R-GQDs has been evaluated via Raman spectroscopy, as shown in Figure S3. Two characteristic peaks have appeared at 1354 cm^−1^ (D band) and 1556 cm^−1^ (G band), indicating the presence of both sp^2^ and sp^3^ hybridized carbon within the R-GQDs [28]. The intensity ratio of the D band to the G band (I_D_/I_G_) has been measured at 0.98, reflecting relatively high crystallinity. These findings have confirmed that the synthesis conditions were successfully optimized through response surface methodology, leading to the effective preparation of N,S co-doped R-GQDs.

### 2.4. Optical Properties of R-GQDs

**Figure 3:**
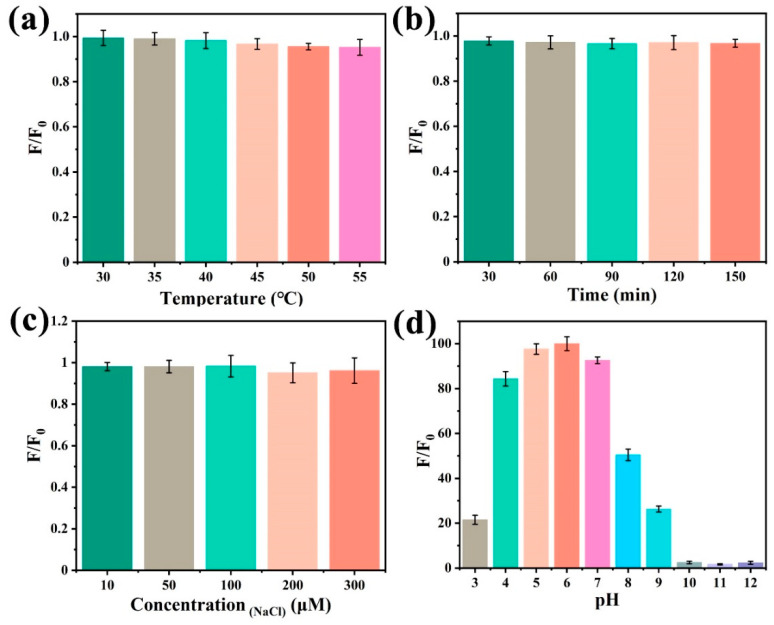
Effects of different conditions on R-GQDs fluorescence, (**a**) temperature; (**b**) anti-photobleaching time; (**c**) NaCl concentration; (**d**) pH. The values of F and F_0_ represent the measured fluorescence values of R-GQDs and the fluorescence values of R-GQDs measured under different conditions (ethanol: water, 30:70, *v*/*v*). Different F/F_0_ values have been marked with various colors in the figure.

The optical properties of R-GQDs under various complex environmental conditions have been further examined by recording their fluorescence spectra. The fluorescence emission behavior of R-GQDs has been evaluated under different temperatures, varying NaCl concentrations, and prolonged UV light exposure. As illustrated in Figure 3a, when the temperature increased from 30 °C to 55 °C, the fluorescence intensity of R-GQDs slightly decreased; however, the emission efficiency has remained above 90%. The fluorescence emission of R-GQDs has shown remarkable thermal stability, attributed to the robust π-π conjugated system, the presence of abundant amine, hydroxyl, and thiol functional groups, as well as the high degree of crystallinity. Given the significant temperature variations encountered in organic solvent matrices, this stability is highly beneficial for detecting water content in organic solvents.

The photobleaching resistance of R-GQDs was subsequently evaluated. As shown in Figure 3b, after continuous irradiation with a handheld UV lamp (365 nm) for 150 min, the fluorescence intensity of the R-GQDs remained virtually unchanged. This remarkable photostability can be attributed to the stable electronic structure of the π-π conjugated system and the highly ordered sp^2^ carbon atom arrangement within the R-GQDs. These properties make R-GQDs highly suitable for long-term water content detection in organic solvents.

In addition, we also studied the salt resistance and pH stability of R-GQDs in an ethanol/water mixed solution of 30:70 (*v*/*v*) to explore its potential applicability under high ionic strength and different pH conditions. As shown in Figure 3c, even at a NaCl concentration of up to 300 μM, R-GQDs still maintained strong fluorescence emission efficiency. Finally, we detected the fluorescence intensity of R-GQDs under different pH conditions at an excitation wavelength of 536 nm. As shown in Figure 3d, R-GQDs exhibited enhanced fluorescence stability under neutral and weakly acidic conditions. This stability is likely attributed to the protonation and deprotonation of amino and thiol groups on the surface of R-GQDs under extreme pH conditions, which leads to changes in the surface charge distribution and thus affects the fluorescence properties [29].

### 2.5. Detection of Water Content in Organic Solvents

Before conducting water content analysis in organic solvents, the anti-interference ability of R-GQDs has been investigated to evaluate the influence of potential interferents, such as metal ions or small molecules, on the sensor’s performance. As shown in Figure S4a, R-GQDs have exhibited a fluorescence response only to Mn^2+^ and certain amino acids that are slightly soluble in ethanol, while the effects of other interfering ions have been negligible. Given the low concentrations of Mn^2+^ and other amino acid molecules in organic solvents, the coexistence of R-GQDs with these interferents in organic solvents is not expected to affect the stability of the fluorescence emission during detection.

The solvent selectivity of R-GQDs was further examined. As shown in Figure S4b, only water significantly affected the fluorescence of R-GQDs, making it a key solvent in altering their emission properties. When polar protic solvents such as ethylene glycol, methanol, ethanol, and water were used, no notable redshift in fluorescence emission was observed as solvent polarity increased, though fluorescence quenching occurred. In contrast, polar aprotic solvents, including tetrahydrofuran, ethyl acetate, acetone, acetonitrile, dimethyl sulfoxide, and N,N-dimethylformamide, exhibited a clear redshift in fluorescence emission with increasing polarity, accompanied by quenching. This solvent-dependent fluorescence behavior of R-GQDs highlights their responsiveness to solvent properties, underscoring their potential as practical sensors for water content detection in organic solvents.

**Figure 4:**
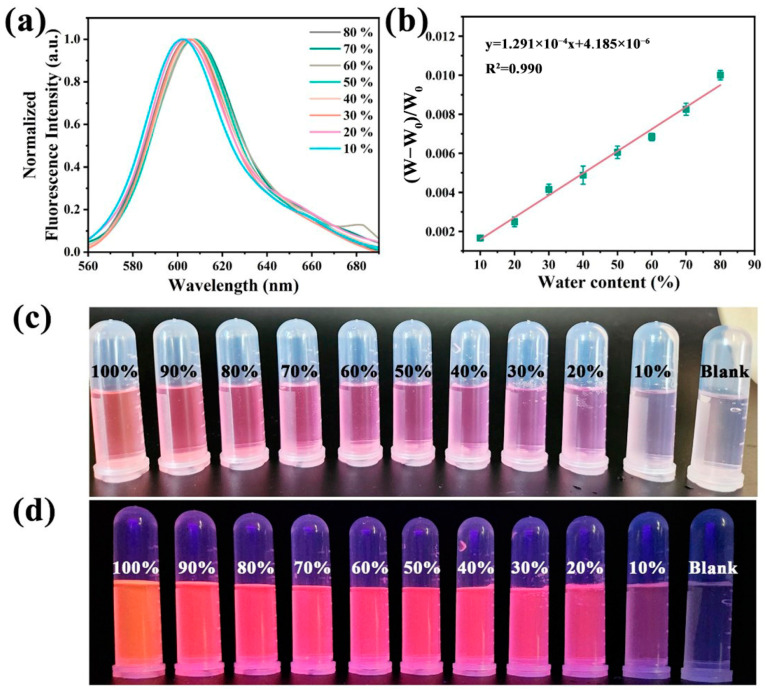
(**a**) Fluorescence emission curve of R-GQDs detecting water content in DMF; (**b**) fitting curve and image of R-GQDs during moisture content detection; (**c**) sunlight; (**d**) fluorescence.

To assess the potential of R-GQDs for detecting water content in organic solvents, their fluorescence spectra have been analyzed in THF, DMF, and ethanol with varying water concentrations. As shown in Figure 4a, the fluorescence emission shift ((W − W_0_)/W_0_) in DMF has exhibited a gradual redshift from 602 nm to 608 nm with increasing water content. A strong linear correlation (R^2^ = 0.990) has been observed between the emission peak position and water content, ranging from 10% to 80% (Figure 4b), with a low detection limit (LOD) of 0.065%. Additionally, the solution’s color under daylight has shifted from orange-yellow to pink and eventually pale purple with increasing water content (Figure 4c). Under 365 nm UV light, the fluorescence has transitioned from orange to magenta, followed by gradual quenching (Figure 4d). This fluorescence color change provides a promising method for visually monitoring the water content in organic solvents, especially R-GQDs are almost applicable in the full concentration range from 0 to 100% (*v*/*v*) which highlighting the great potential of R-GQDs in practical applications.

**Figure 5:**
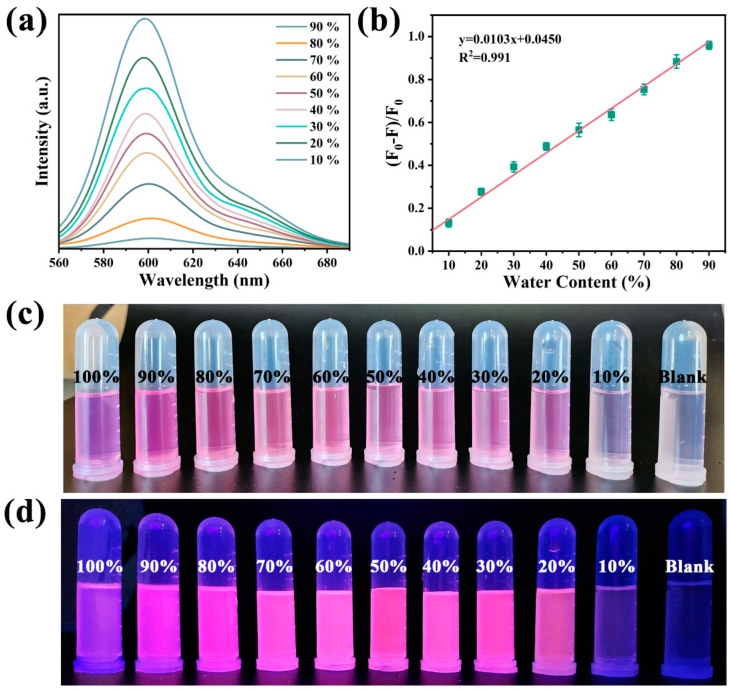
(**a**) Fluorescence emission curve of R-GQDs detecting water content in THF; (**b**) fitting curve and image of R-GQDs during moisture content detection; (**c**) sunlight; (**d**) fluorescence.

For water content detection in THF, the fluorescence emission intensity of R-GQDs has progressively decreased with increasing water concentration, accompanied by a slight redshift (Figure 5a). A strong linear relationship between fluorescence intensity and water content has been observed as the water concentration has increased from 10% to 80% (R^2^ = 0.991) (Figure 5b), with a detection limit (LOD) of 0.070%. Concurrently, the solution color has shifted from purple to a lighter magenta, becoming nearly colorless and transparent at 80% water content (Figure 5c). Under 365 nm UV illumination, the fluorescence has transitioned from purple to pale pink, followed by quenching (Figure 5d).

**Figure 6:**
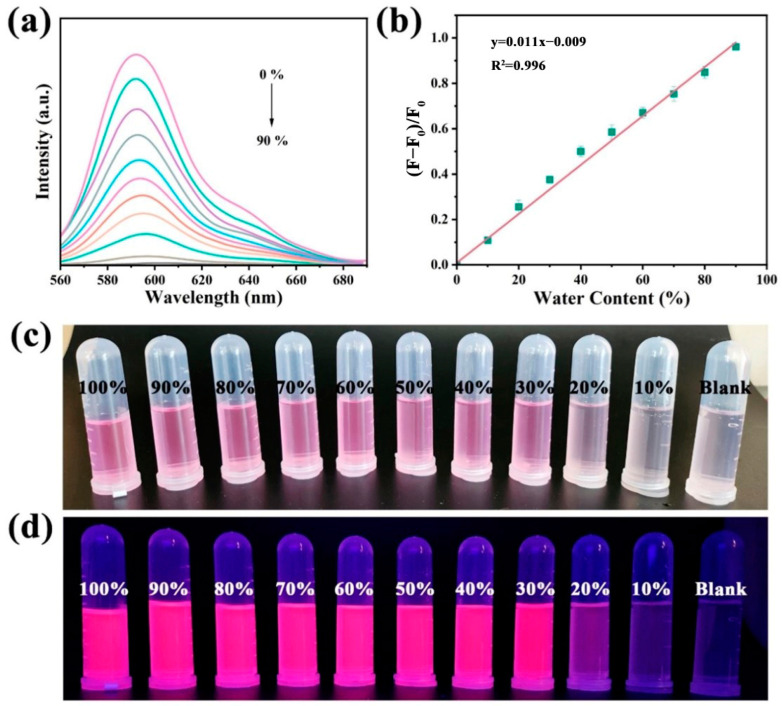
(**a**) Fluorescence emission curve of R-GQDs detecting water content in ethanol; (**b**) fitting curve and image of R-GQDs during moisture content detection; (**c**) sunlight; (**d**) fluorescence.

For water content detection in ethanol, even at water concentrations as high as 90%, the solvent has displayed residual fluorescence. Within the range of 10% to 90% water content, the fluorescence intensity of R-GQDs has gradually decreased with increasing water content (Figure 6a). A strong linear correlation has been observed between fluorescence intensity and water content (R^2^ = 0.996), with a limit of detection (LOD) of 0.066%, calculated using the 3σ/k method (Figure 6b). Under daylight, the solution color has faded from pink to colorless (Figure 6c), and under 365 nm UV light, the fluorescence has transitioned from bright magenta to full quenching (Figure 6d). Table 1 compared the reported sensors for detecting water in organic solvents, and the results showed that R-GQDs had a wider linear range and higher sensitivity than other sensors.

These results have demonstrated that R-GQDs are capable of detecting water content in DMF, THF, and ethanol with high sensitivity and almost full concentration linear range detection from 0 to 100% (*v*/*v*). This has proven R-GQDs to be a promising material for real-time monitoring of water content in organic solvents. Furthermore, their excellent accuracy and selectivity have highlighted their potential for practical applications in water content detection in both industrial and laboratory environments.

### 2.6. Response Mechanism for Detecting Water Content

**Figure 7:**
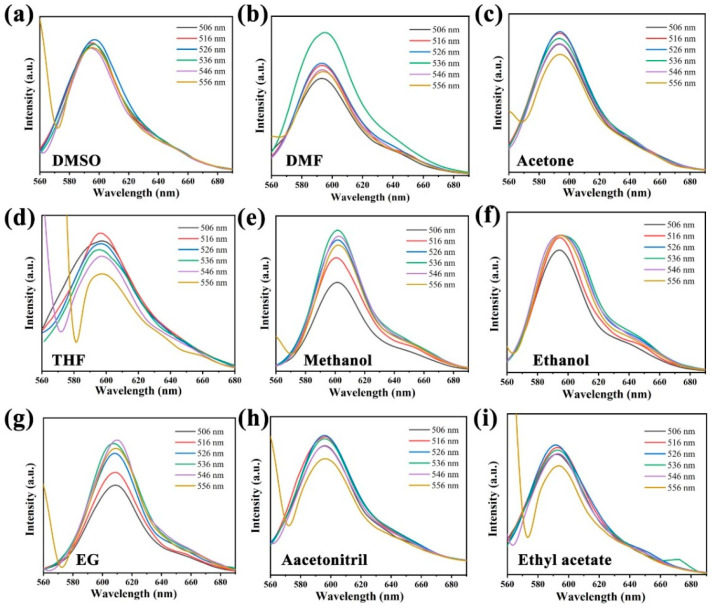
(**a**) Fluorescence spectra of R-GQDs in (**a**) DMSO; (**b**) DMF; (**c**) acetone; (**d**) THF; (**e**) methanol; (**f**) ethanol; (**g**) EG; (**h**) acetonitrile; (**i**) ethyl acetate.

When examining the underlying factors responsible for fluorescence spectral shifts, it has been essential to consider the electron donor-acceptor properties and the solute-solvent interactions influenced by solvent polarity. Fluorescence spectra collected for R-GQDs across different organic solvents have consistently demonstrated excitation-independent emission characteristics in all tested environments (Figure 7).

Furthermore, the solvent-dependent behavior of R-GQDs has been described using the Reichardt polarity parameter ($E_{T}^{N}$), a standardized metric that accounts for a solvent’s overall solvating ability, including non-specific interactions (e.g., dipole-dipole interactions, DD) and specific interactions (e.g., hydrogen bonding) [29]. As shown in Table S5, in polar aprotic solvents, as the $E_{T}^{N}$ value of the solvent has increased, the fluorescence emission peak of R-GQDs has gradually shifted toward longer wavelengths. This increase in Stokes shift has resulted in a significant decrease in absolute fluorescence quantum yield (QY). In contrast, in polar protic solvents, the fluorescence emission peak and absolute fluorescence quantum yield of R-GQDs have remained relatively unchanged. Therefore, the solvent-dependent behavior has been attributed to the nature of the organic solvents, as the amino and thiol groups on the surface of R-GQDs have shown sensitivity to solvent polarity. Changes in solvent polarity may have caused a redshift, blueshift, or quenching of the fluorescence emission. Moreover, excitation independence and solvatochromism have been characteristic of the molecular state in GQDs, confirming that the fluorescence emission of R-GQDs originates from molecular-state emission.

The photoluminescence of R-GQDs has originated from n-π* and π-π* transitions, where the n-orbital has served as the highest occupied molecular orbital (HOMO) due to the negative inductive effect [32]. As illustrated in Figure S5, the fluorescence emission peak of R-GQDs has undergone a gradual redshift with increasing polarity of aprotic solvents. Concurrently, absorption bands at 542 nm and 580 nm have shifted towards longer wavelengths as solvent polarity has risen (Figure S6). This phenomenon has occurred because the increasing solvent polarity has influenced the negative inductive effect, stabilizing the excited electrons by modulating the dipole moment orientation of surrounding molecules. As solvent polarity has intensified, the negative inductive effect has strengthened, enhancing the capacity for dipole moment adjustment in surrounding molecules, which has altered the electron density distribution within the R-GQDs, narrowed the energy gap, and induced redshifts in both the UV and fluorescence spectra, thereby manifesting the solvatochromic behavior of R-GQDs [33,34].

**Figure 8:**
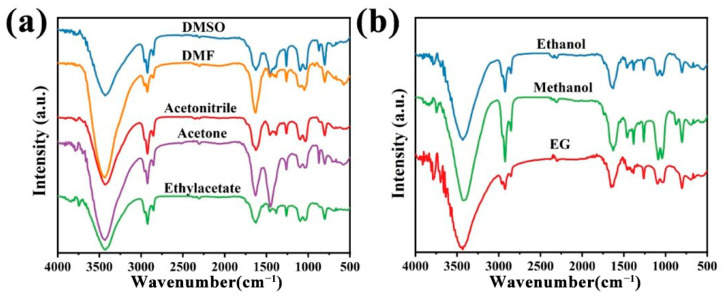
FT-IR spectra of freeze-dried or vacuum-dried graphene quantum dots dispersed in aprotic solvents (**a**) and protic solvents (**b**).

In contrast, the emission wavelength of R-GQDs in protic solvents has remained almost unchanged despite variations in solvent polarity. For instance, although ethylene glycol exhibits significantly higher polarity than ethanol, the fluorescence emission shift is merely 6 nm. Thus, while the emission wavelength shift in protic solvents is influenced by solvent polarity, it is less dependent on polarity compared to aprotic solvents. This suggests that the fluorescence quenching of R-GQDs in protic solvents likely arises from hydrogen bonding interactions, a conclusion corroborated by the observed shifts in characteristic peaks of amino and thiol groups on the surface of R-GQDs in different solvents, as indicated in the FT-IR spectra (Figure 8).

### 2.7. Actual Sample Analysis

To assess the feasibility and accuracy of R-GQDs as a fluorescent probe for detecting water content in real samples, a spiking recovery method has been employed for evaluating water content in liquor and alcoholic beverages. Utilizing the fitted linearship y = 0.011x − 0.009, the recovery rates have been calculated to range from 90% to 114.40%, with a relative standard deviation (RSD) of less than 2.78 (Table 2). These findings indicate that R-GQDs exhibit significant potential for detecting water content in ethanol/H_2_O systems.

### 2.8. Visual Inspection Test Strips

The measurement of water content in organic solvents through simple, rapid, and efficient methods has been a significant research focus. While the solvent-dependent chromatic response of R-GQDs facilitates visual detection, the human eye’s ability to discern subtle color changes lacks sufficient accuracy. Leveraging the excellent fluorescence stability of R-GQDs, we have developed them into a colorimetric test strip for portable water content sensing with smartphone assistance. By coating R-GQDs onto commercially available filter paper, we created a portable sensor, which, in combination with a handheld UV lamp, dark box, and smartphone, forms an effective detection platform for measuring water content in organic solvents.

**Figure 9:**
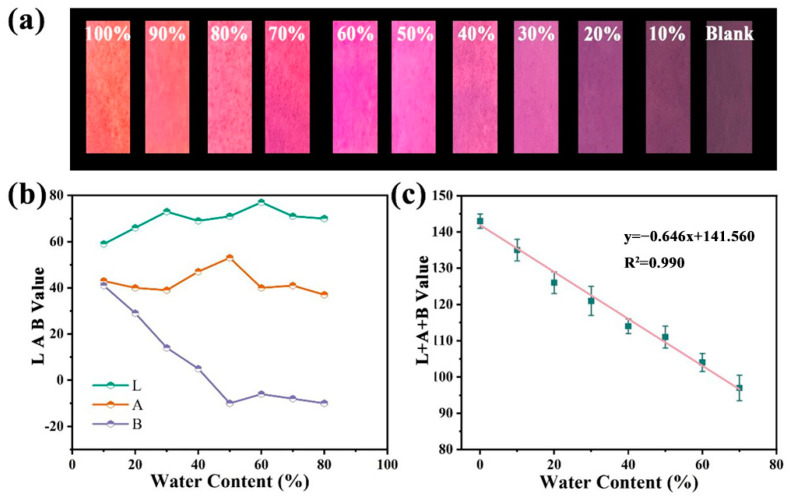
(**a**) Images of test strips with varying water contents in DMF taken under 365 nm UV light; (**b**) trends of L, A and B values of R-GQDs test strips with changing water content; (**c**) linear relationship between the sum of L, A and B values and water content. Images of the test paper taken under 365nm ultraviolet light when testing different water contents in DMF.

The LAB color model, which simulated the human visual system, has been recognized for its significant advantages over the traditional RGB model, particularly in terms of color difference calculation, cross-device color conversion, and color correction and optimization. The LAB color model, which provided more nuanced color gradients than RGB and CMYK models, enhanced color analysis recognition. In the LAB model, L represents brightness, A indicated the magenta-green spectrum, and B signified the yellow-blue range. Therefore, we have utilized Colorsnap software to analyze the color parameters of the samples under investigation. As illustrated in Figure 9a, upon application of the sample to the test strip, the strip’s color transitioned from yellow to pink and ultimately to deep red as water content in DMF increased. The variations in L, A, and B values in response to changing water content are depicted in Figure 9b, where the B value decreases with increasing water content, while the A value exhibits a slower decline. Figure 9c shows that a linear relation-ship has been established by fitting the sum of L, A, and B values with water content (0–70%): y = −0.646x + 141.560 (R^2^ = 0.990), with corresponding color data and the relationship between water content and R + G + B values are listed in Figure S7 and Table S6.

**Figure 10:**
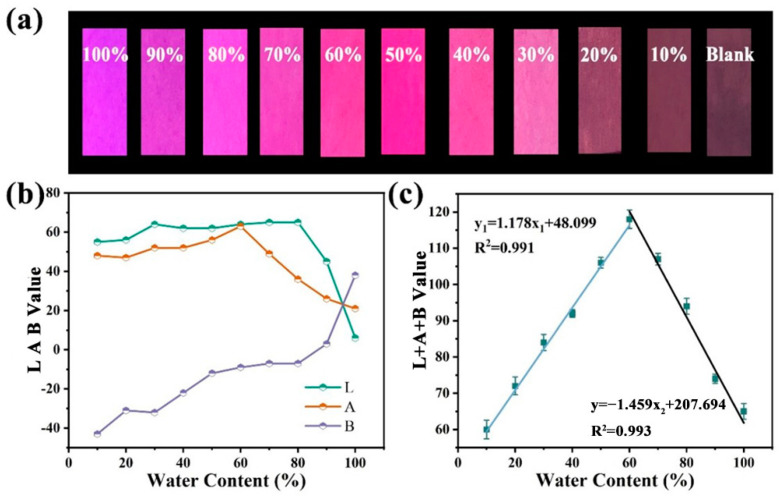
(**a**) Images of test strips with varying water contents in THF taken under 365 nm UV light; (**b**) trends of L, A and B values of R-GQDs test strips with changing water content; (**c**) linear relationship between the sum of L, A and B values and water content.

Additionally, the water content in tetrahydrofuran (THF) has been assessed. As shown in Figure 10a, applying THF with varying water content levels to the test strip resulted in a color transition from purple to fuchsia and ultimately to dark red. Figure 10b illustrates the variations in L, A, and B values of the test strip in response to changes in water content. Figure 10c presents the linear relationships between the sum of L, A, and B values and water content for the ranges of 0–60% and 60–100%, expressed as: y_1_ = 1.178x_1_ + 48.099 (for 10–60%) and y_2_ = −1.459x_2_ + 207.694 (for 60–100%), with R^2^ values of 0.991 and 0.993, respectively. The corresponding color data and the relationship between water content and R + G + B values are listed in Figure S8 and Table S7.

**Figure 11:**
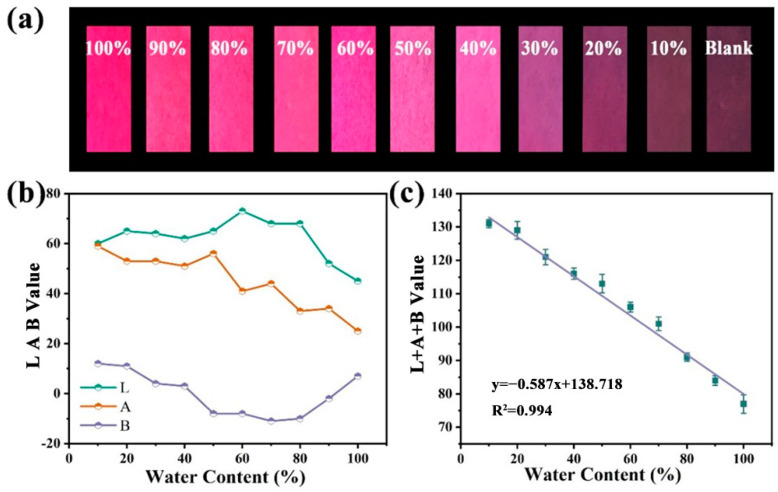
(**a**) Images of test strips with different water contents taken under 365 nm UV light; (**b**) trends of L, A and B values of R-GQDs test strips with changing water content; (**c**) linear relationship between the sum of L, A and B values and water content.

The water content in the ethanol solvent has been analyzed. As shown in Figure 11a, applying ethanol with varying water content to the test strip resulted in no significant color change; instead, the fuchsia fluorescence gradually quenched to dark red. The L value of the test strip exhibited minimal variation, while the A and B values decreased significantly (Figure 11b). The linear relationship between the sum of L, A and B values and the water content (0–100%) was: y = −0.587x + 138.718 (R^2^ = 0.994), as illustrated in Figure 11c. The corresponding color data and the relationship between water content and R + G + B values are listed in Figure S9 and Table S8.

### 2.9. In Vivo Fluorescence Imaging of Freshwater Shrimp

**Figure 12:**
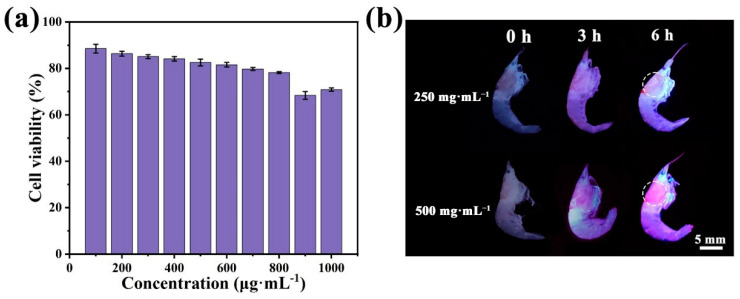
(**a**) Cell survival rate of N-GQDs at different concentrations for 24 h; (**b**) Fluorescence field images of shrimp at different culture times.

The bioimaging capability of R-GQDs has been evaluated using in vivo imaging in small animal models. Prior to this application, MTT assays conducted with 4T1 cells indicated that at a concentration of 1000 μg·mL^−1^, cell viability remained above 70% after 24 h of incubation (Figure 12a). This demonstrates that the R-GQDs synthesized in this study exhibit low biotoxicity within the concentration range of 0–1000 μg·mL^−1^. Additionally, the potential of R-GQDs for in vivo imaging was explored by treating black tiger shrimp with R-GQDs. Juvenile shrimp were incubated in dispersions of R-GQDs at concentrations of 250 mg·mL^−1^ and 1000 mg·mL^−1^ for varying durations (0, 3, and 6 h). Following irradiation with a 365 nm UV lamp, images were captured using a smartphone (Figure 12b). Notably, as the incubation time increased, R-GQDs gradually accumulated in the visceral region while preserving the shrimp’s external morphology and mobility. These findings indicate that R-GQDs possess good biocompatibility and safety, suggesting their potential as fluorescent imaging probes for in vivo applications.

## 3. Materials and Methods

Materials and reagents section, instrumentation details and determination of water content detection were provided in Supplementary Materials.

### 3.1. Synthesis of R-GQDs

To synthesize R-GQDs, 0.23 g of o-phenylenediamine was dissolved in 20 mL of a sulfuric acid/water (*v*:*v* = 1:19) mixed solution. The solution was then transferred into a 50 mL Teflon-lined stainless-steel autoclave and heated at 200 °C for 5 h. After the reaction mixture was cooled to room temperature, the solution was neutralized to pH 7 using 1.0 M NaOH. The black solid was completely precipitated, and the resulting mixture was filtered through a 0.22 μm filter to collect the solid product. The product was then dried in a vacuum oven at 60 °C for subsequent use.

To achieve R-GQDs with optimal emission intensity, the synthesis conditions were optimized using a standard Box-Behnken Design (BBD). A Box-Behnken Response Surface Methodology (BBD-RSM) with four factors and three levels was employed to design the experiments. The response surface experimental data were processed using the Design Expert 8.0.6 software. Four independent variables were considered: the concentration of o-phenylenediamine (X_1_), the volume ratio of sulfuric acid/water (X_2_), the reaction temperature (X_3_), and the reaction time (X_4_). Based on preliminary experiments, all variables were set at three levels (−1, 0, 1), and a second-order equation was employed to optimize the experiment. The Equation (2) is shown as:

(2) $$Y=\beta_{0}+\Sigma_{i=1}^{k}\beta_{i}X_{i}+\Sigma_{i=1}^{k}\beta_{ii}X_{i}^{2}+\Sigma_{i=1}^{k}\Sigma_{j>1}^{k}\beta_{ij}X_{i}X_{j}$$

where Y represents the predicted fluorescence intensity, Xi and Xj are the independent variables, β_0_ denotes the intercept, and β_i_, β_ii_, and β_ij_ are the linear, quadratic, and interaction coefficients, respectively.

### 3.2. Control of Water Content in Solvents

Water was artificially added to anhydrous solvents to determine whether the water content in each anhydrous solvent can be effectively controlled.

### 3.3. Preparation and Data Statistics of Colorimetric Test Paper Based on R-GQDs

Utilizing the excellent solubility of R-GQDs in both organic solvents and water, R-GQDs were employed as a dye for fabricating portable sensors. Briefly, commercial filter paper was cut into 1 cm × 3 cm strips and subsequently immersed in a 2 mg·mL^−1^ ethanol solution of R-GQDs for 5 s. The soaked filter paper was then air-dried at room temperature until all water contant had completely evaporated, resulting in a portable sensor strip. After drying, 50 μL of the test liquid was added to the surface of the sensor strip, which was then placed in a dark chamber equipped with a UV lamp. Under 365 nm UV irradiation, the fluorescence images of the test strips were captured using a smartphone. The water content was analyzed using the ColorSnap color analysis software.

### 3.4. Cytotoxicity

The in vitro cytotoxicity of N-GQDs was evaluated using the standard MTT assay. 4T1 cells were seeded into culture dishes and incubated for 24 h in Dulbecco’s Modified Eagle Medium (DMEM) supplemented with 10% fetal bovine serum and 1.0% anti-mycoplasma antibiotics, under standard conditions of 37 °C and 5.0% CO_2_. Subsequently, the cells were exposed to varying concentrations of R-GQDs (0–1 mg·mL^−1^) in DMEM for an additional 24 h. After incubation, 10 μL of MTT solution (5 mg·mL^−1^) was added to each well, followed by a 4 h incubation period to allow the formation of purple formazan crystals. The supernatant was carefully removed, and 200 μL of DMSO was added to each well to dissolve the formazan crystals by shaking the plate for 10 min on a microplate shaker [35]. Finally, the optical density (OD) was measured at 490 nm using a microplate reader, and the cytotoxicity was calculated based on Equation (3).

(3) $$Cell\;viability\%=\frac{Sample\;absorbance-blank\;sample\;absorbance}{Absorbance\;of\;control\;group-absorbance\;of\;blank\;sample}\times 100\%$$

### 3.5. In Vivo Fluorescence Imaging

For imaging experiments, freshwater shrimp were cultured in a water dispersion of R-GQDs containing 20% ethanol for varying time periods (0, 3, and 6 h). After treatment, the shrimp were removed and placed onto a clean glass slide. The slide containing the treated shrimp was then placed in a dark box equipped with 365 nm UV illumination. Visual fluorescence images of the N-GQDs-treated shrimp under UV excitation were captured using a smartphone camera.

## 4. Conclusions

In this work, sulfur and nitrogen co-doped red fluorescent graphene quantum dots (R-GQDs) were successfully synthesized using a one-pot hydrothermal method with o-phenylenediamine as the carbon source in an H_2_SO_4_/water solution. The synthesis conditions were optimized using response surface methodology. The R-GQDs exhibited excellent fluorescence stability and were capable of dual-mode detection (colorimetric and fluorescence) of water content in organic solvents, leveraging solvent polarity and dipole-dipole interactions. In addition to a wide linear detection range and high sensitivity, the R-GQDs were coated onto paper strips to serve as a portable paper-based sensor. The color changes on the strips were quantified by converting them into LAB values using a smartphone, enabling precise water content quantification in organic solvents. Further validation was carried out through spiked recovery experiments in food samples, confirming the practical applicability of R-GQDs as water content sensors. Additionally, the low background fluorescence and good biocompatibility of R-GQDs facilitated their use in in vivo imaging of freshwater shrimp. Overall, this study provides a novel real-time water content detection strategy for organic solvents and highlights the potential of R-GQDs in biological imaging applications.

## Figures and Tables

**Table 1:** Comparison of R-GQDs detection of water content in organic solvents with other work.

Type of Sensor	Line Range (μM)		LOD (%)		Ref.
N-CDs/PAA	1.000–30.000%	EtOH	0.071%	EtOH	[30]
	0.200–13.000%	Actone	0.027%	Actone	
o-CDs	0–0.800%	DMF	0.200%	DMF	[31]
	0–0.800%	THF	0.100%	THF	
CDs	0.100–2.500%	Actone	0.460%	Actone	[19]
R-GQDs	10.000–80.000%	DMF	0.065%	DMF	This Work
	10.000–80.000%	THF	0.070%	THF	
	10.000–90.000%	EtOH	0.066%	EtOH	

**Table 2:** Determination of water content in food matrices.

Sample	Added (%)	Found (%)	Recovery (%)	RSD (%) *n* = 3
alcoholic beverages	10.00	9.34	90.00	1.03
	20.00	21.96	109.8	0.42
	40.00	39.75	99.38	1.59
	60.00	64.38	107.3	2.18
Liquor	10	11.44	114.40	2.78
	20	23.39	116.96	1.47
	40	41.54	103.85	0.64
	60	61.33	102.24	1.91

## Data Availability

Data are contained within the article.

## Supplementary Materials

The following supporting information can be downloaded at: https://www.mdpi.com/article/10.3390/molecules29215178/s1, Figure S1 (a) Actual and predicted fluorescence intensity; (b) Residual and predicted fluorescence intensity; Figure S2 Three-dimensional response surface plot: (a) reaction time and amount of o-phenylenediamine; (b) reaction time and reaction temperature; (c) reaction time and sulfuric acid/water volume ratio; (d) reaction temperature and o-phenylenediamine amount; (e) sulfuric acid/water volume ratio and o-phenylenediamine amount; (f) reaction temperature and sulfuric acid/water volume ratio; Figure S3 Raman spectrum of R-GQDs; Figure S4 Anti-interference ability of R-GQDs in detecting water content in organic solvents; Figure S5 Normalized fluorescence spectra of R-GQDs dispersed in (a) aprotic solvent and (b) protic solvent; Figure S6 Normalized absorption spectra of R-GQDs dispersed in (a) aprotic solvent and (b) protic solvent; Figure S7 Relationship between water content and R+G+B value in DMF; Figure S8 Relationship between water content and R+G+B value in THF; Figure S9 Relationship between water content and R+G+B value in ethanol; Table S1 Factors in response surface analysis; Table S2 Experimental factors and results of synthesizing R-GQDs using BBD-RSM design; Table S3 Analysis of variance of regression model; Table S4 Statistical analysis of regression model errors; Table S5 $E_{N}^{T}$ values, emission peak positions and fluorescence quantum yields in different solvents; Table S6 Color parameters of the test strips for detecting moisture content in DMF obtained using a smartphone APP; Table S7 Color parameters of the test strips for detecting moisture content in THF obtained using a smartphone APP; Table S8 Color parameters of the test strips for detecting moisture content in ethanol obtained using a smartphone APP.
