# Supplementary material for: Nitrogen and Sulfur Co-Doped Graphene-Quantum-Dot-Based Fluorescent Sensor for Rapid Visual Detection of Water Content in Organic Solvents
Source: Molecules. 2024 Nov 1;29(21):5178. doi: 10.3390/molecules29215178 (PMC11547257; doi:10.3390/molecules29215178)
Supplement: Supplementary file 1 [file molecules-29-05178-s001.zip › molecules-3265311-supplementary.pdf]

Nitrogen and Sulfur Co-Doped Graphene-Quantum-Dot-Based Fluorescent Sensor for Rapid Visual Detection ofWater Content in Organic Solvents

Hongyuan Zhang 1,*, Jieqiong Wang 2, Xiaona Ji 1, Yanru Bao 1, Ce Han 3,* and Guoying Sun 4,*

1 School of Science, Changchun Institute of Technology, 395 Kuanping Road, Changchun 130012, China; lx_jxn@ccit.edu.cn (X.J.); 024baoyanru@163.com (Y.B.)

2 School of Materials Science and Engineering, Changchun University, 6543, Weixing Road, Changchun 130022, China; wangjq94@ccu.edu.cn

3 State Key Laboratory of Electroanalytical Chemistry, Changchun Institute of Applied Chemistry, Chinese Academy of Sciences, Changchun 130022, China

4 School of Chemistry and Life Science, Changchun University of Technology, 2055 Yanan Street, Changchun 130012, China

* Correspondence: zhanghongyuan@ccit.edu.cn (H.Z.); hance@ciac.ac.cn (C.H.); sunguoying@ccut.edu.cn (G.S.)

Reagents and materials

o-phenylenediamine, sulfuric acid, sodium hydroxide, methanol, acetone, ethanol, acetonitrile, anhydrous N,N-dimethylformamide (DMF), dimethyl sulfoxide (DMSO) ethyl acetate and ethylene glycol (EG) , were bought from Macklin (China). Ultrapure water prepared by Milli-Q Gradient ultrapure water system (Millipore) was used throughout the experiments.

Instrument

The morphological and crystallographic characteristics of the materials were investigated by high-resolution transmission electron microscope (HR-TEM) (JEOL-2100F). UV-Vis spectra were measured on a spectrophotometer (Varian Cary 50). Fourier transform infrared spectroscopy (FT-IR) measurements were carried out using Is50 (PerkinElmer, USA). Raman spectra were used to analyze the structure information of the as-obtained samples (LABRAM HR Evolution). X-ray diffraction (XRD) patterns of the samples were measured using Cu Kα radiation (RIGAKU D MAX 2500). X-ray photoelectron spectroscopy (XPS) was performed using monochromated Al Kα radiation as an X-ray source (A VG ESCALAB MKII spectrometer). Fluorescence excitation and emission spectra were collected with a fluorescence spectrometer (PerkinElmer LS-55). All photographs of cells were captured by an inverted fluorescence microscope (DMI4000B, Leica). The absolute photoluminescence quantum yield (Φ) was measured with QuantaMaster 8000 fluorescence spectrometer.

Fluorescence detection of water content in organic solvents

In a typical detection procedure, 1.5 mg of R-GQDs was dispersed into organic solvents (DMF, THF, and ethanol) containing different amounts of water. The resulting mixtures were sonicated for 2 minutes to ensure thorough dispersion of the samples. Subsequently, the fluorescence emission spectra were recorded under an excitation wavelength of 536 nm. For each experiment, fluorescence spectra were collected three times to calculate the relative standard deviation (RSD) and evaluate the accuracy of the experimental data. The limit of detection (LOD) was calculated using the formula 3σ/K

where σ represents the standard deviation of the blank sample, and K is the slope of the curve within the linear response range.

Table S1 Factors in response surface analysis

Factors	Units	Factor Levels		
		-1	0	+1
A-time(X_1)	h	1.00	5.00	9.00
B-temperature(X_2)	℃	180	200.00	220.00
C- H_2SO_4/water(X_3)	%	0.00	5.00	10.00
D- concentration(X_4)	mg·mL^{-1}	1.00	11.50	22.00

Table S2 Experimental factors and results of synthesizing R-GQDs using BBD-RSM design

Group	Level				Fluorescence Intensity
	X_1	X_2	X_3	X_4	
1	0	200	10	22	689.73
2	1	200	10	11.5	708.11
3	5	200	5	11.5	746.39
4	5	200	5	11.5	746.39
5	5	200	10	1	703.44
6	5	200	5	11.5	746.39
7	5	180	5	22	732.79
8	1	180	5	11.5	589.07
9	1	200	5	22	703.44
10	1	220	5	11.5	684.65
11	5	180	10	11.5	683.74
12	5	180	5	1	679.78
13	9	200	0	11.5	602.17
14	5	200	5	11.5	746.39
15	5	200	5	11.5	746.39
16	9	200	10	11.5	681.18
17	5	200	0	1	702.30
18	9	200	5	1	700.84

Group	Level				Fluorescence Intensity
	X_1	X_2	X_3	X_4	
19	1	200	5	1	689.73
20	9	220	5	11.5	727.16
21	5	200	0	22	675.43
22	9	200	5	22	704.07
23	1	200	0	11.5	630.88
24	9	180	5	11.5	689.24
25	5	220	5	1	699.68
26	5	220	5	22	704.07
27	5	220	10	11.5	689.42
28	5	220	0	11.5	710.46
29	5	180	0	11.5	737.60

Table S3 Analysis of variance of regression model

Source of variance	Sum of squares	Degrees of freedom	Mean square	F value	P value
Model	40677.58	22	1848.98	4.58	0.0333
A-time	34.46	1	34.46	0.09	0.7801
B-temperature	19.45	1	19.45	0.05	0.8336
C- H_2SO_4/water	59.60	1	59.60	0.15	0.7141
D- concentration	411.68	1	411.68	1.02	0.3517
AB	831.17	1	831.17	2.06	0.2014
AC	0.79	1	0.79	1.96×10^{-3}	0.9661
AD	27.46	1	27.46	0.068	0.8030
BC	268.96	1	268.96	0.67	0.4457
BD	590.98	1	590.98	1.46	0.2719
CD	43.30	1	43.30	0.11	0.7545
A^2	14738.53	1	14738.53	36.49	0.0009
B^2	2733.93	1	2733.903	6.77	0.0406
C^2	7801.31	1	7801.31	19.31	0.0046
D^2	1151.28	1	1151.28	2.85	0.1423
Residual	2423.74	6	403.96		
Lack of fit	2423.74	2	1211.87		
Net error	0	4	0		
Total deviation	43101.32	28			

Table S4 Statistical analysis of regression model errors

Project statistics	Value
Standard deviation	20.10
Mean	698.31
Coefficient of variation CV%	2.88
Sum of squares of prediction error	0.3495
Determination coefficient R^2	0.9438
Adjustment coefficient R^2	0.7376
Prediction coefficient R^2	-7.0976
Model precision Press	8.4730

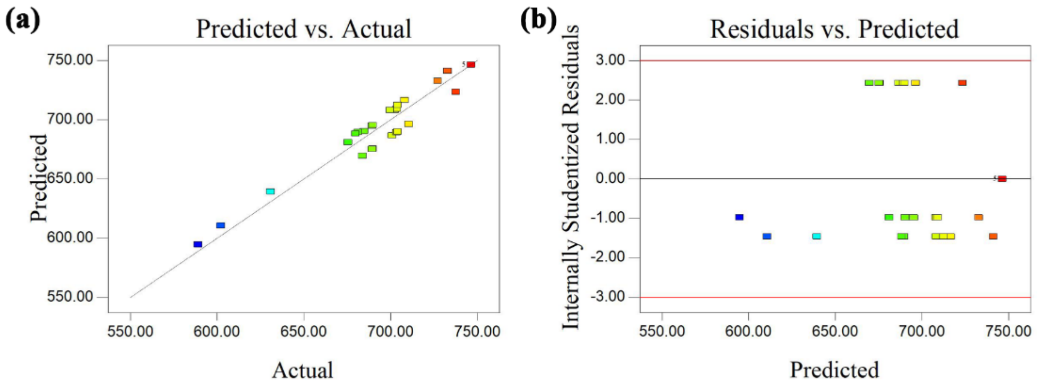

Figure S1 (a) Actual and predicted fluorescence intensity; (b) Residual and predicted fluorescence intensity

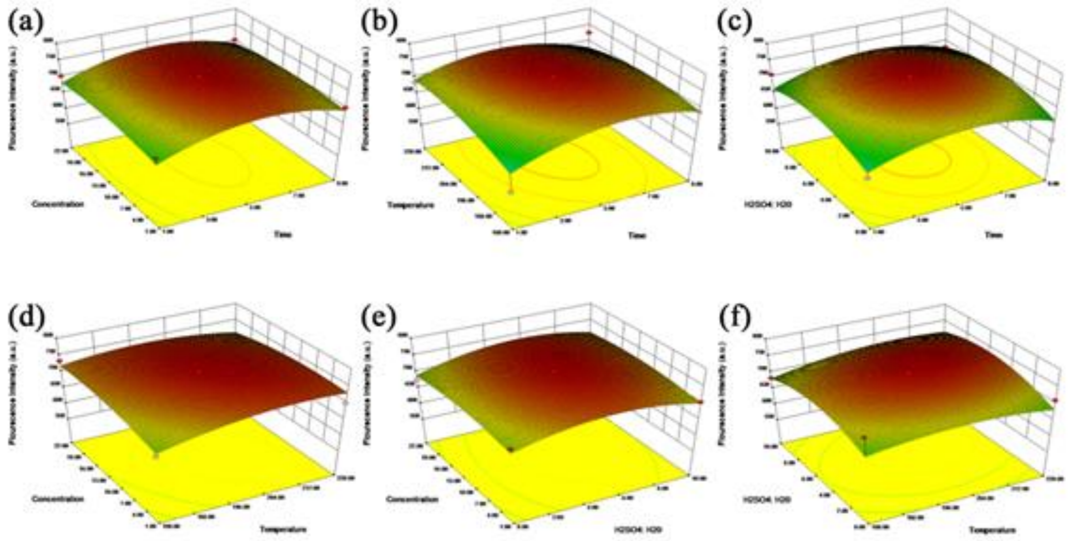

Figure S2 Three-dimensional response surface plot: (a) reaction time and amount of o-phenylenediamine; (b) reaction time and reaction temperature; (c) reaction time and sulfuric acid/water volume ratio; (d) reaction temperature and o-phenylenediamine amount; (e) sulfuric acid/water volume ratio and o-phenylenediamine amount; (f) reaction temperature and sulfuric acid/water volume ratio

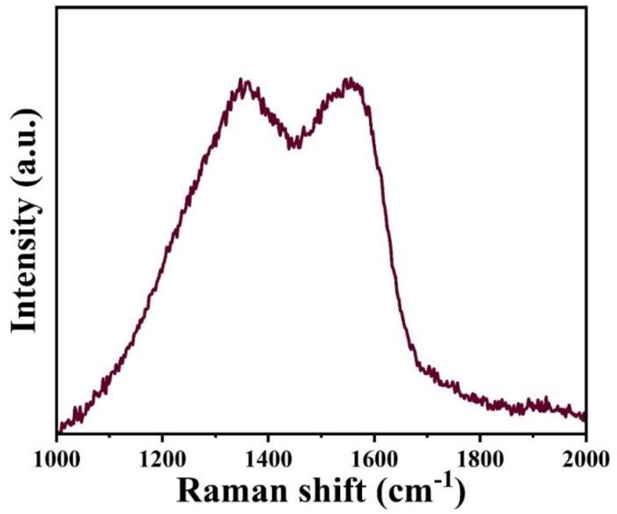

Figure S3 Raman spectrum of R-GQDs

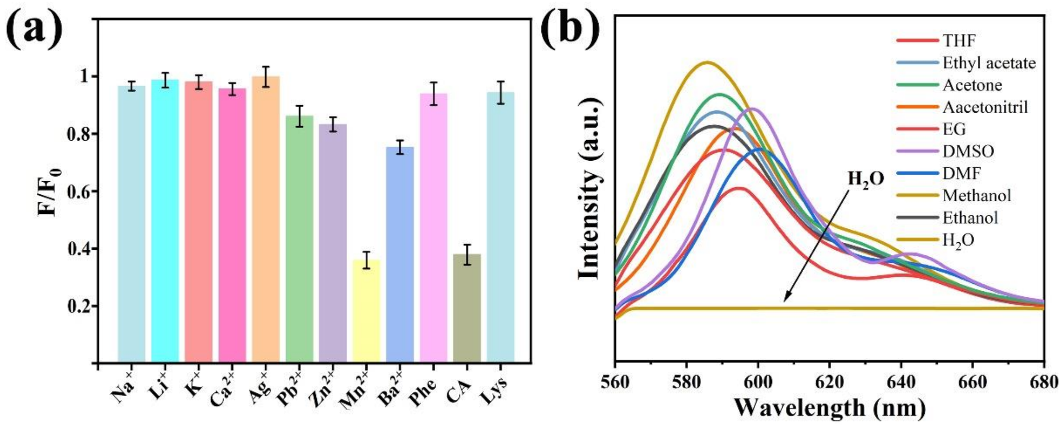

Figure S4 Anti-interference ability of R-GQDs in detecting water content in organic solvents

Table S5 E_N^Tvalues, emission peak positions and fluorescence quantum yields in different solvents

Solvent	E_N^T	λ_{max} (nm)	PLQY (%)
THF	0.207	601	34.38
Ethyl acetate	0.228	590	28.35
Acetone	0.355	595	24.38
Acetonitrile	0.460	595	17.35
DMF	0.386	592	16.98
DMSO	0.444	598	17.35
Ethanol	0.654	596	12.80
Methanol	0.762	601	9.65
EG	0.790	602	5.87

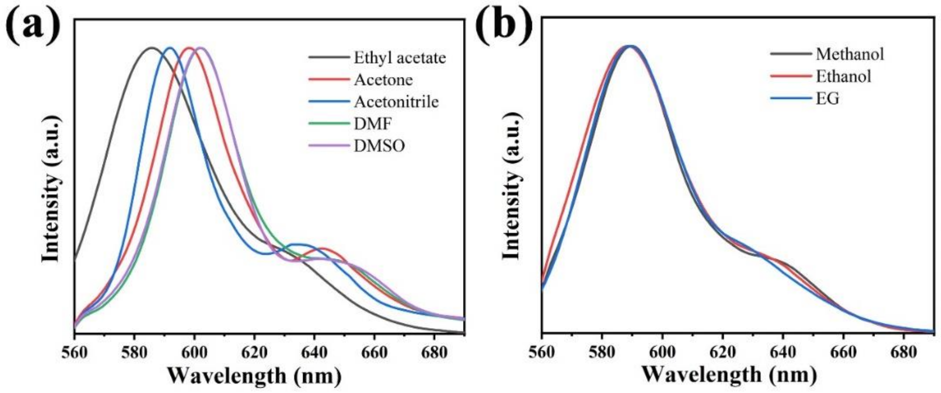

Figure S5 Normalized fluorescence spectra of R-GQDs dispersed in (a) aprotic solvent and (b) protic solvent

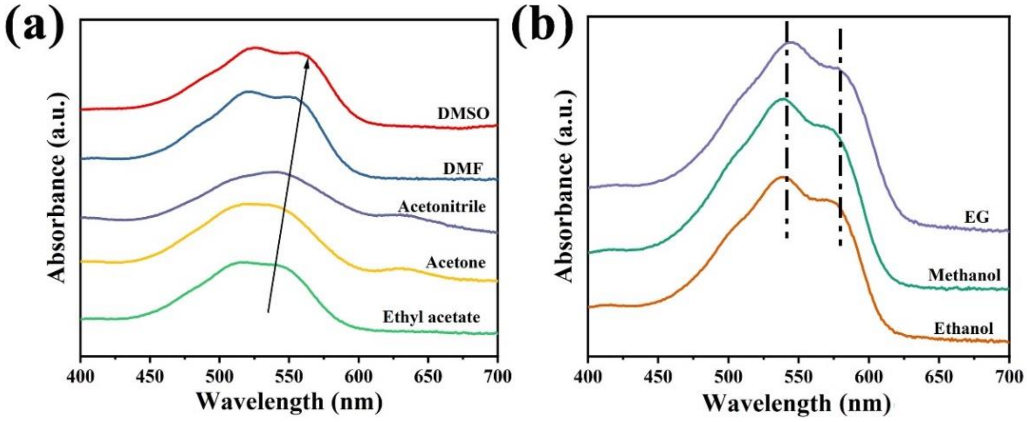

Figure S6 Normalized absorption spectra of R-GQDs dispersed in (a) aprotic solvent and (b) protic solvent

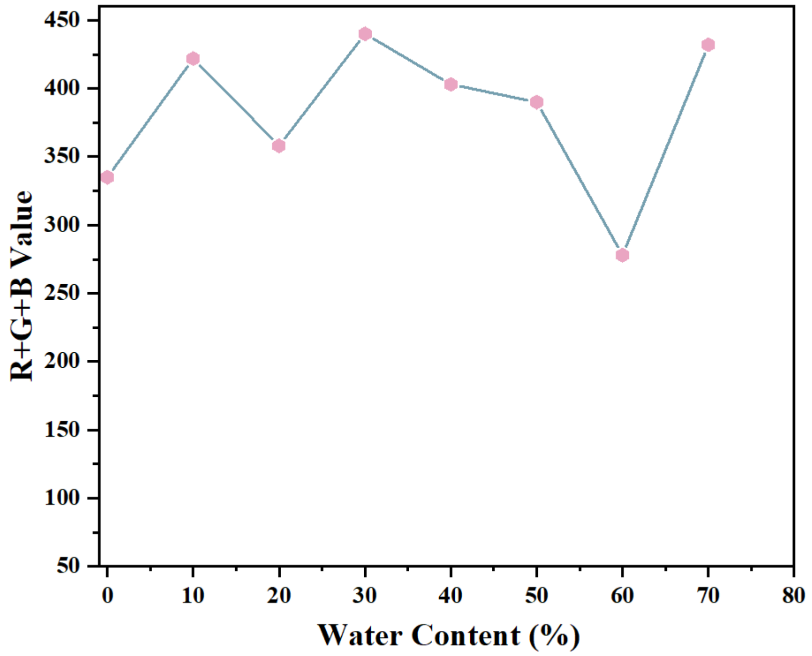

Figure S7 Relationship between water content and R+G+B value in DMF

Table S6 Color parameters of the test strips for detecting moisture content in DMF obtained using a smartphone APP

Color	Water content (%)	R value	G value	B value	L value	A value	B value	L+A+B value
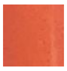	0	195	78	62	59	43	41	143
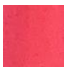	10	230	87	105	66	40	29	143
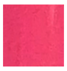	20	206	54	98	73	39	14	135
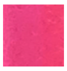	30	231	77	132	69	47	5	126
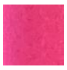	40	211	71	121	71	53	-10	121
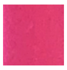	50	214	65	111	77	40	-6	114
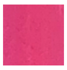	60	109	60	109	71	41	-8	111
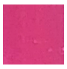	70	213	86	133	70	37	-10	104

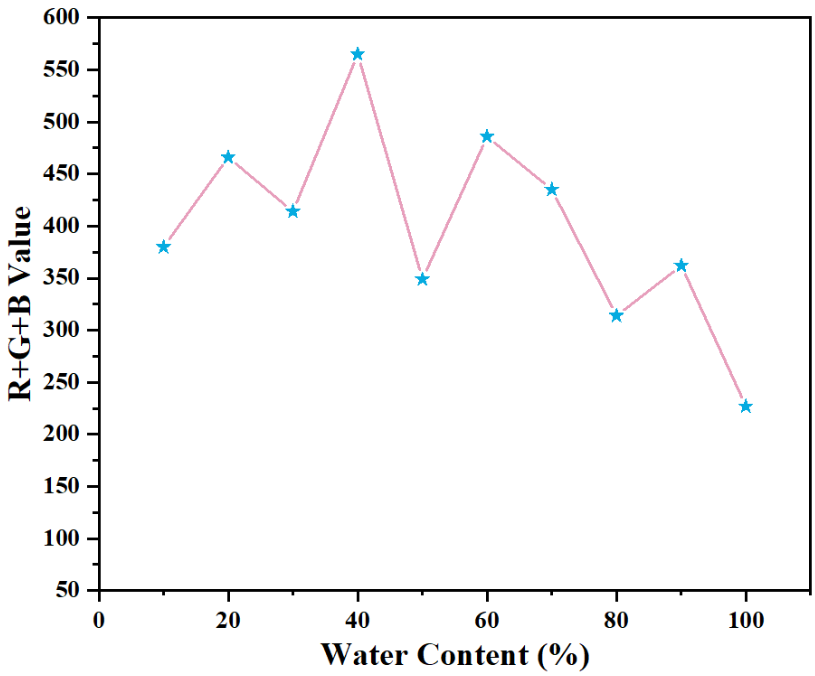

Figure S8 Relationship between water content and R+G+B value in THF

Table S7 Color parameters of the test strips for detecting moisture content in THF obtained using a smartphone APP

Color	Water content (%)	R value	G value	B value	L value	A value	B value	L+A+B value
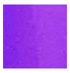	10	119	39	222	55	48	-43	60
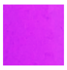	20	221	27	218	56	47	-31	72
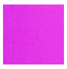	30	193	36	185	64	52	-32	84
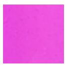	40	239	113	213	62	52	-22	92
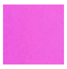	50	179	28	142	62	56	-12	106
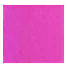	60	224	65	197	64	63	-9	118
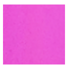	70	209	65	161	65	49	-7	107
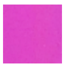	80	150	29	135	65	36	-7	94
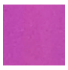	90	164	46	152	45	26	3	74
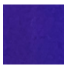	100	64	42	121	6	21	38	65

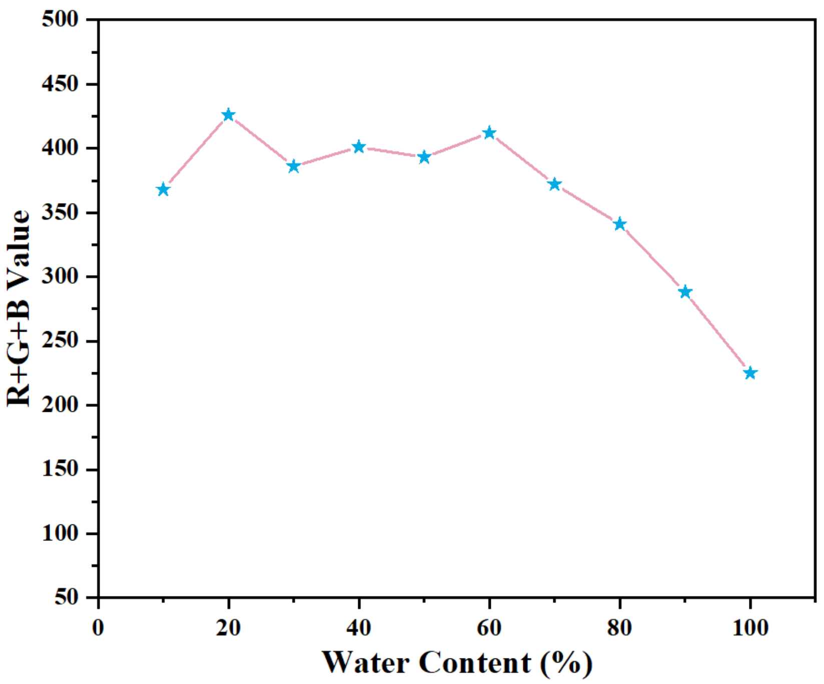

Figure S9 Relationship between water content and R+G+B value in ethanol

Table S8 Color parameters of the test strips for detecting moisture content in ethanol obtained using a smartphone APP

Color	Water content (%)	R value	G value	B value	L value	A value	B value	L+A+B value
	10	212	31	125	60	59	12	131
	20	227	45	154	65	53	11	129
	30	225	29	132	64	53	4	121
	40	222	41	138	62	51	3	116
	50	225	29	139	65	56	-8	113
	60	238	31	143	73	41	-8	106
	70	227	18	127	68	44	-11	101
	80	217	8	116	68	33	-10	91
	90	110	48	130	52	34	-2	84
	100	65	52	108	45	25	7	77
